# Effects of cafeteria diet and caloric restriction on pituitary hormones and metabolic phenotype in male Wistar rats

**DOI:** 10.1007/s12020-025-04448-9

**Published:** 2025-10-15

**Authors:** Christakis Kagios, Susanne Hetty, Fleur Hukema, Giovanni Fanni, Erika Roman, Jan W Eriksson

**Affiliations:** 1 Clinical Diabetology and Metabolism, Uppsala University, Uppsala, Sweden; 2 Department of Pharmaceutical Biosciences, Uppsala University, Uppsala, Sweden; 3 Department of Animal Biosciences, Swedish University of Agricultural Sciences, Uppsala, Sweden

**Keywords:** Calorie restriction, Diet-induced obesity, MCSF, Metabolism, Obesity, Pituitary hormones

## Abstract

**Purpose:**

Obesity is associated with neuroendocrine and metabolic dysregulation, yet the underlying mechanisms remain incompletely understood. This study aimed to investigate how pituitary hormonal axes and peripheral hormones respond to a cafeteria diet or a calorie-restricted diet in rats.

**Methods:**

Ten-week-old male Wistar rats (*n* = 36) were randomized (1:1:1) to one of three diets for 12 weeks: an ad libitum standard rat chow diet (control group); an ad libitum cafeteria diet, containing cheese doodles, chocolate balls and salted peanuts, in addition to standard chow (diet-induced obesity group, DIO); or calorie-restriction (aiming at 85% body weight of controls; restricted group). We assessed endocrine gland weights, plasma levels of pituitary hormones and related peripheral signals, and explored their associations with metabolic and behavioral outcomes.

**Results:**

While the DIO group exhibited increased body weight, insulin resistance, and altered metabolic markers, only modest changes in pituitary hormones were observed, with a reduction in luteinizing hormone (*p* < 0.05). Correlation analysis showed that when combining the control and DIO groups, prolactin inversely correlated with exploratory-activity (rho = -0.458, *p* < 0.05) from the behavioral test. In contrast, the restricted group showed more pronounced hormonal changes, including reduced levels of adrenocorticotropic hormone (*p* < 0.01), prolactin, and thyroid-stimulating hormone (both *p* < 0.05) as well as insulin-like growth factor-1 (*p* < 0.01). Multivariate data analysis showed a clear separation of the DIO group from the other groups, mainly driven by metabolic variables.

**Conclusion:**

Despite notable metabolic perturbations in the DIO group, the absence of endocrine changes suggests a partly different phenotype than what is typically observed in humans with obesity.

**Supplementary Information:**

The online version contains supplementary material available at 10.1007/s12020-025-04448-9.

## Introduction 

Over the past decades, the prevalence of obesity has been increasing dramatically resulting in pandemic proportions [1]. This is worrying, as obesity is highly associated with numerous comorbidities, including the metabolic syndrome and type 2 diabetes (T2D), cardiovascular disease and also mental health disorders [2, 3]. The so-called Westernized diet, characterized by a high intake of fat and sugar, is known to lead to weight gain, as well as metabolic and hormonal dysregulation [4]. However, genetic, environmental and lifestyle factors also play an important role [5, 6]. Understanding how this type of diet affects hormonal signaling is important in the fight against obesity, as hormones play a key role in regulating appetite, energy balance, and glucose metabolism.

Both human [7–9] and animal [8, 10, 11] studies have shown that the brain plays an important role in the regulation of energy and glucose turnover, by modulating the autonomic nervous system and several endocrine axes. In humans, the hypothalamus-pituitary hormonal axes, such as the hypothalamic-pituitary-adrenal (HPA) axis, the hypothalamic-pituitary-thyroid (HPT) axis, the hypothalamic-pituitary-gonadal (HPG) axis and the hypothalamic-pituitary-growth hormone (HPGH) axis, regulate not only energy and glucose homeostasis, but also other important processes such as growth, reproductive functions and stress responses [12]. These neuroendocrine pathways are conserved among vertebrate species [8, 13–15], allowing the translation of findings from animals to humans. In rodents, the pituitary gland is divided into the anterior and neurointermediate lobes. Herein, focus is mainly on the anterior pituitary (AP) since it is of much interest in the development of obesity and T2D, as dysregulation of AP hormonal systems has been associated with obesity, metabolic syndrome and diabetes [16–18]. Key hormones such as growth hormone (GH), adrenocorticotropic hormone (ACTH), thyroid stimulating hormone (TSH), luteinizing hormone (LH), follicle-stimulating hormone (FSH), and prolactin are secreted from the AP.

Cafeteria diets have been extensively used to induce obesity and dysglycemia in animal models [19]. By mimicking the Westernized diet and human overconsumption of palatable foods, cafeteria diets can provide an appropriate model to better understand metabolic and behavioral perturbations involved in the development of obesity and T2D [19]. Previous studies have demonstrated that cafeteria diets can increase adiposity and dysregulate glucose and lipid metabolism in animals [20–22]. However, results regarding behavioral assessments have been far from consistent [19, 21]. On the other hand, a calorie-restricted diet, which reduces energy intake, has been shown to have beneficial effects on body weight and insulin sensitivity [21, 23], and also to increase the lifespan of the animals [24, 25]. However, despite the metabolic benefits of caloric restriction, the literature is contradictory in behavioral changes, reporting negative mood regulation [26], improved cognitive recovery and social cooperation [27, 28] or no changes [21, 29]. Thus, there is a need for further exploration of the connection between metabolic, hormonal and behavioral responses after these diets.

Our research group has previously established a rat model with diet induced obesity (DIO), achieved by a high-energy cafeteria diet. These animals developed mild metabolic perturbations and prediabetes, but they did not display any major behavioral changes [21]. We also included a group receiving calorie-restricted diet. In the present work we performed further analyses on the same animals. The aim was to evaluate how the dietary manipulations influenced the activity of pituitary-to-periphery hormonal axes and to examine the interplay between hormonal, metabolic and behavioral functions. We primarily investigated the effects of a high-energy cafeteria diet compared to controls, while the calorie-restricted group was included for exploratory analyses.

## Materials and methods 

### Animals and experimental design

All animal experiments were approved by the Uppsala Animal Ethical Committee (permit number 5.8.18–12996/2022) and followed the guidelines of the Swedish Legislation on Animal Experimentation (Animal Welfare Act SFS 2018:1192) and the European Union Directive on the Protection of Animals Used for Scientific Purposes (Directive 2010/63/EU).

The experimental design has been described previously [21]. In short, 36 male Wistar rats were randomized into three groups: control, cafeteria diet-induced obesity (DIO), and calorie-restricted (*n* = 12/group) for 12 weeks. The control group had access to standard rat chow (ssniff Spezialdiäten GmbH, Germany) ad libitum. The DIO group had ad libitum access to both standard rat chow and an ad libitum cafeteria diet consisting of chocolate balls, cheese doodles, and roasted, salted peanuts. The restricted group was maintained at around 85% of the body weight of the control animals by adjusting the amount of standard rat chow that they received. All animals had access to water ad libitum. Directly after euthanasia by brief anesthesia followed by decapitation, trunk blood was collected and centrifuged to separate and store the plasma at -80 °C until further analysis. Endocrine organs (pituitary gland, divided into anterior and neurointermediate lobes, adrenals, and testes) were dissected out and weighed.

Other metabolic assessments and the behavioral test using the multivariate concentric square field™ (MCSF) have been described previously [21]. The amount of dopamine and its receptors, a key catecholamine neurotransmitter system involved in reward, motivation, and stress regulation, has also been implicated in metabolic processes relevant to obesity and type 2 diabetes, which are also reported elsewhere [30].

As previously reported [21], the mean body weights after 12 weeks of diet differed between the groups, 485.0 g, 574.8 g, and 424.2 g, respectively, for the control, DIO, and restricted groups (*p* < 0.001 vs. control). The DIO group had higher insulin and glucose levels compared to the control and restricted groups, and they displayed insulin resistance as reflected by the homeostatic model assessment for insulin resistance (HOMA-IR; 24.95, 36.90, and 21.55 for the control, DIO, and restricted groups, respectively).

### Measurement of hormones and other biomarkers

Plasma ACTH and corticosterone levels were simultaneously quantified using the Milliplex^®^ MAP Rat Stress hormone magnetic bead panel (#RSHMAG-69 K; EMD Millipore, Billerica, MA, United States).

Other plasma pituitary hormones (FSH, GH, LH, TSH, and prolactin) and, in addition, brain-derived neurotrophic factor (BDNF) levels were simultaneously quantified using the Milliplex^®^ Rat Pituitary magnetic bead panel (#RPTMAG-86 K; EMD Millipore, Billerica, MA, United States).

Plasma glucagon levels were quantified using the Milliplex^®^ MAP Rat Metabolic expanded magnetic bead panel (#RMHE-120 K; EMD Millipore, Billerica, MA, United States).

Plasma triiodothyronine (T3) and thyroxine (T4) levels were quantified using the Milliplex^®^ Rat Thyroid Hormone magnetic bead panel (#RTHYMAG-30 K; EMD Millipore, Billerica, MA, United States).

The Milliplex^®^ assays were performed according to the manufacturer’s instructions. In short, all analyses were run in duplicates in 96-well plates. The Bio-Plex 200 (Bio-Rad, CA, USA) was used to obtain and analyze the results by measuring the Median Fluorescent Intensity (MFI). Standard curves were generated, and two Quality Control samples with known concentrations were included to ensure accuracy. The minimum limits of detection for ACTH and corticosterone were 0.5 pg/mL and 1796 pg/mL, respectively, both with an intra- and inter-assay variation of less than 10%. For FSH, LH, TSH, prolactin and BDNF the minimum limits of detection were 7.62 pg/mL, 3.28 pg/mL, 0.87 pg/mL, 4.64 pg/mL and 0.38 pg/mL, respectively, all with an intra-assay variation of less than 10% and inter-assay variation less than 15%. The minimum limits of detection for GH and glucagon were 6.50 pg/mL and 4.0 pg/mL, respectively, with an intra-assay variation of less than 10% and inter-assay variation of less than 20%. Lastly, the minimum limits of detection for T3 and T4 were 195 pg/mL and 2321 pg/mL, respectively, with an intra-assay variation of less than 15% and inter-assay variation of less than 10%.

Quantification of plasma testosterone was performed by the enzyme-linked immunosorbent assay (ELISA) Testosterone Parameter™ Assay (KGE010, R&D Systems, Minneapolis, MN, USA) kit in accordance with the manufacturer’s protocol, with a mean minimum detectable concentration of 0.03 ng/mL.

Quantification of plasma IGF-1 was performed by the ELISA Quantikine™ Rat IGF-1 Immunoassay (MG100, R&D Systems, Minneapolis, MN, USA) kit in accordance with the manufacturer’s protocol, with a mean minimum detectable concentration of 3.5 pg/mL.

Quantification of plasma C-peptide was performed by the Rat C-Peptide ELISA (10-1172-01, Mercodia, Uppsala, Sweden) kit in accordance with the manufacturer’s protocol, with a minimum detectable concentration of 99 pmol/L.

Quantification of plasma copeptin was performed by the Rat Copeptin ELISA (NBP3-42433, R&D Systems, Minneapolis, MN, USA) kit in accordance with the manufacturer’s protocol, with a mean minimum detectable concentration of 9.37 pg/mL.

### Statistical analyses

Data are expressed as mean ± standard deviation (SD) or median and interquartile range (IQR) as appropriate according to distribution. Normality was assessed using the Shapiro-Wilk W test. Outliers were removed after applying the Grubbs test and confirming non-physiological values through separate evaluation. Given that our primary objective was to investigate the effects of a high-energy cafeteria diet compared to controls, with the calorie-restriction group included for exploratory purposes, group comparisons were conducted using t-tests for normally distributed data, and the Mann-Whitney U test for non-normally distributed data. Since not all data were normally distributed, Spearman correlations were used to explore the relationship between pituitary hormones with previously published metabolic and behavioral parameters [21], and dopamine levels in specific brain regions [30]. Data were considered statistically significant at *p* < 0.05. All statistical analyses were carried out in IBM SPSS Statistics version 28 software and in GraphPad Prism 10, with the latter being used also for figure creation.

An orthogonal partial least squares discriminant analysis (OPLS-DA) was performed to examine group differences based on diet and to highlight which variables of the pituitary hormones and previously published metabolic parameters [21], most strongly associated with these differences. The software SIMCA (version 18.0.1, Sartorius Stedim Data Analytics AB, Umeå, Sweden) was used for the analysis.

## Results

### Weight of endocrine glands

Weights of endocrine glands are shown in Table 1. Pituitary gland weight was significantly lower in both the DIO and restricted groups in comparison to the control (*p* < 0.01; *p* < 0.001, respectively). However, only the DIO group had significantly lower (*p* < 0.001) relative pituitary gland weight when adjusting for body weight. When dividing the pituitary gland into the anterior and neurointermediate lobes, the same results were observed for the anterior lobe as for the whole pituitary gland. The absolute weight was significantly lower in both the DIO and restricted groups (*p* < 0.01; *p* < 0.001, respectively), while only the DIO group (*p* < 0.001) had significantly lower relative anterior lobe weight compared to the control group. The neurointermediate lobe weight was significantly lower for the restricted group (*p* < 0.05) in comparison to controls. However, when adjusted for body weight, the relative weight was significantly lower only for the DIO group (*p* < 0.01). Regarding the adrenals and testis weights, no differences were found in absolute weights in the DIO and restricted groups compared to controls. However, the relative weights of both the adrenals and testes in the DIO group were significantly lower (*p* < 0.05; *p* < 0.001, respectively) in comparison to controls, whereas they were significantly higher in the restricted group (*p* < 0.05; *p* < 0.01, respectively).

**Table 1:** Weights of endocrine glands (mg or g) and relative tissue weights (mg/g of total body weight) of the pituitary gland, anterior pituitary lobe, neurointermediate pituitary lobe, adrenals and testes in the control, diet-induced obesity (DIO) and restricted groups (*n* = 9–12/group)

Tissue	Units	Control	DIO	Restricted
Pituitary gland	mg	22.6 ± 3.9	17.7 ± 4.0 ##	18.0 ± 1.5 ###
	mg/g BW	0.047 ± 0.008	0.034 ± 0.004 ***	0.043 ± 0.004
Anterior lobe	mg	15.7 ± 3.7	12.1 ± 1.6 ##	12.0 ± 1.3 ###
	mg/g BW	0.032 ± 0.008	0.022 ± 0.002 ###	0.028 ± 0.003
Neurointermediate lobe	mg	6.9 ± 0.9	6.6 ± 0.9	6.1 ± 0.6 *
	mg/g BW	0.014 ± 0.002	0.012 ± 0.002 **	0.014 ± 0.002
Adrenals	mg	63.8 ± 14.3	58.8 ± 13.3	64.2 ± 10.1
	mg/g BW	0.135 ± 0.021	0.113 ± 0.016 *	0.157 ± 0.015 *
Testes	g	4.1 ± 0.2	4.0 ± 0.3	3.8 ± 0.7
	mg/g BW	8.41 ± 0.50	7.01 ± 0.82 ###	8.95 ± 1.35 ##

Data represent mean ± SD

BW, body weight

* p < 0.05, ** p < 0.01, *** p < 0.001 (t-test) and ## p < 0.01, ### p < 0.001 (Mann-Whitney U test) compared to the control group

### Hypothalamic-pituitary-adrenal (HPA) axis

**Fig. 1:**
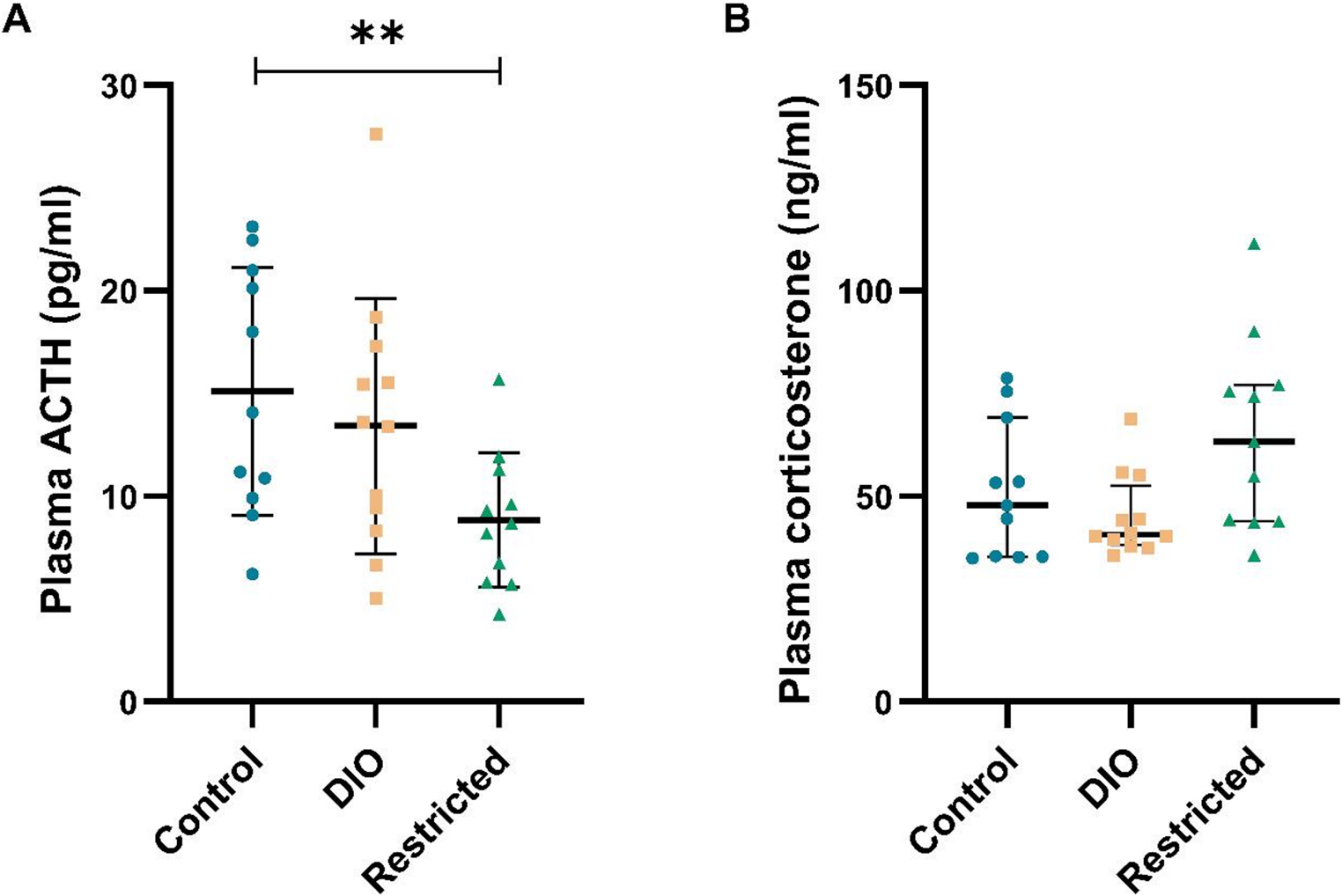
Plasma ACTH **A** and corticosterone **B** levels in rats, after 12 weeks of diet for the control, diet-induced obesity (DIO), and restricted groups (*n* = 11–12/group). Data represent mean ± SD for **A** and median with IQR for **B**. ** *p* < 0.01 compared to the control group (t-test)

ACTH levels were significantly lower in the restricted group compared to the control group (*p* < 0.01; Fig. 1A), while corticosterone levels were unchanged (Fig. 1B). No differences in ACTH and corticosterone levels were observed between the DIO and the control group (Fig. 1).

In the correlation analyses, when combining the control group with the DIO group (Supplementary Table 1), corticosterone correlated positively with dopamine levels in the hypothalamus (rho = 0.671, *p* < 0.05). When instead the control group was combined with the restricted group (Supplementary Table 2), ACTH correlated positively with dopamine levels in the hypothalamus (rho = 0.636, *p* < 0.05).

### Hypothalamic-pituitary-gonadal (HPG) axis

**Fig. 2:**
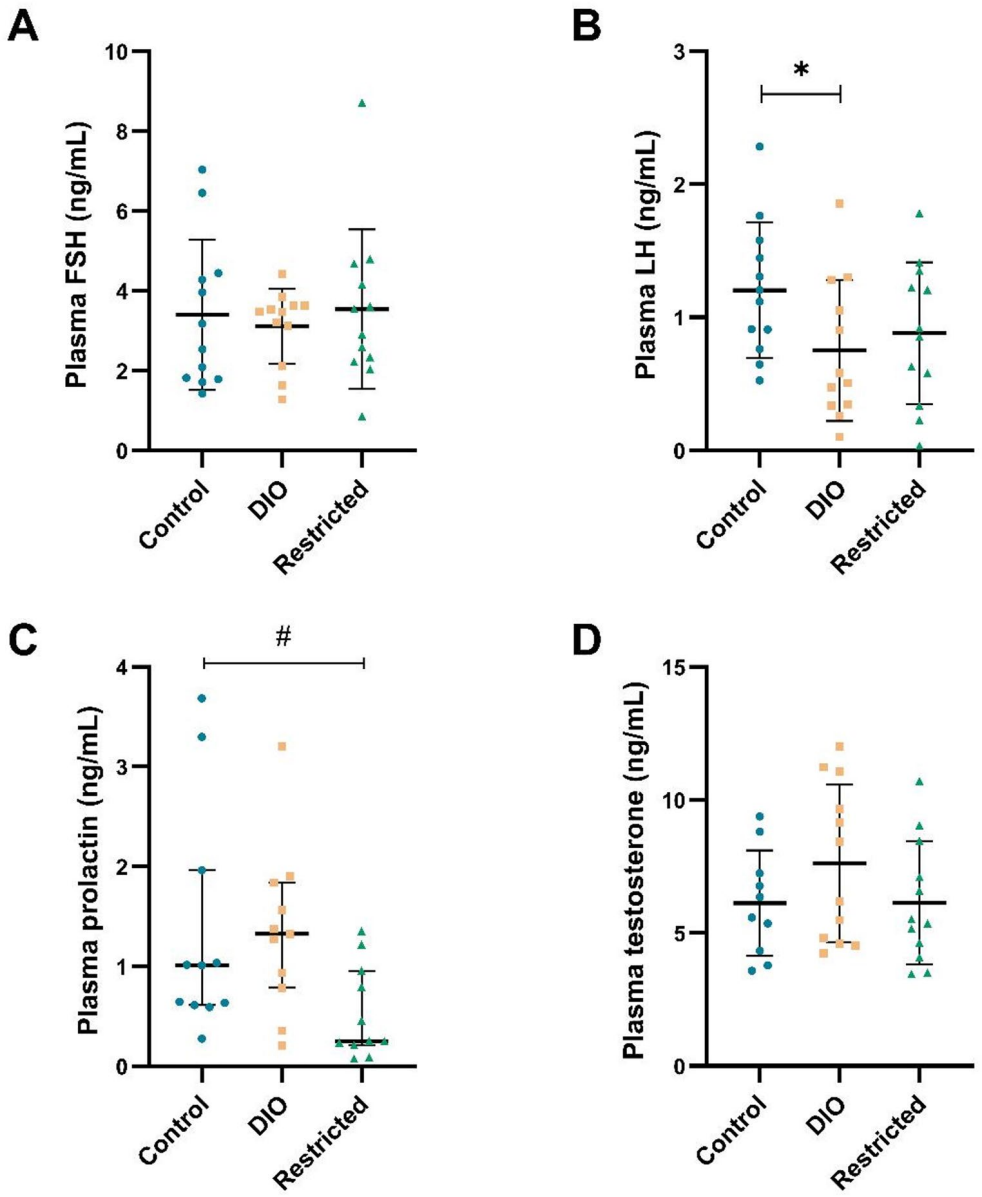
Plasma FSH **A**, LH **B**, prolactin **C**, and testosterone **D** levels in rats, after 12 weeks of diet for the control, diet-induced obesity (DIO), and restricted groups (*n* = 10–12/group). Data represent mean ± SD for **A**, **B**, and **D**, and median with IQR for **C**. * *p* < 0.05 (t-test) and # *p* < 0.05 (Mann-Whitney U test) compared to the control group

No group differences were observed in FSH levels compared to controls (Fig. 2A). LH levels were significantly lower for the DIO group compared to controls (*p* < 0.05, Fig. 2B), while prolactin levels were significantly lower for the restricted group compared to controls (*p* < 0.05, Fig. 2C). Lastly, plasma testosterone levels did not differ between the groups (Fig. 2D).

In the correlation analyses, when combining the control group with the DIO group (Supplementary Table 1), FSH levels was inversely correlated with dopamine levels in the caudate putamen (rho = -0.895, *p* < 0.001). Neither prolactin nor TSH correlated with dopamine levels in any of the analyzed brain regions, including the cingulate cortex, caudate putamen, nucleus accumbens shell and core, and the hypothalamus. LH correlated inversely with risk-taking behavior (rho = -0.495, *p* < 0.05) while prolactin inversely correlated with exploratory activity (rho = -0.458, *p* < 0.05) from the behavioral test. Lastly, testosterone correlated positively with dopamine levels in the nucleus accumbens core (rho = 0.580, *p* < 0.05).

### Growth hormone and thyroid hormones

**Fig. 3:**
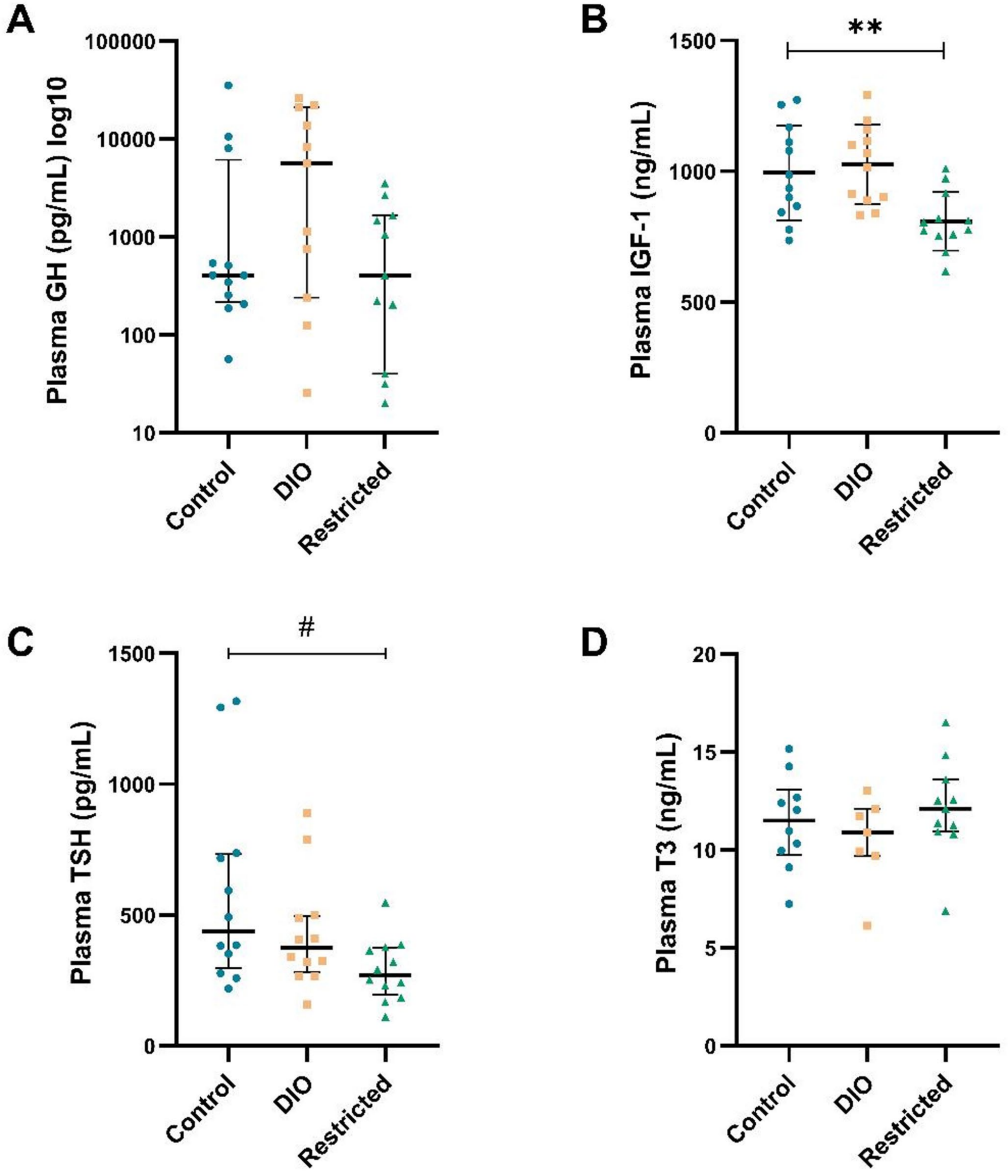
Plasma GH **A** (logarithmic scale), IGF-1 **B**, TSH **C**, and T3 **D** levels in rats, after 12 weeks of diet for the control, diet-induced obesity (DIO), and restricted groups (*n* = 7–12/group). Data represent mean ± SD for **B** and **D**, and median with IQR for **A** and **C**. ** *p* < 0.01 (t-test) and # *p* < 0.05 (Mann-Whitney U test) compared to the control group

No differences in GH, IGF-1, TSH, or T3 levels were observed between the DIO group and the control group (Fig. 3). Moreover, GH levels did not differ between the restricted group and controls (Fig. 3A). However, the restricted group had significantly lower IGF-1 levels (*p* < 0.01, Fig. 3B) and TSH levels (*p* < 0.05, Fig. 3C) compared to controls, while no differences in T3 levels were observed (Fig. 3D). T4 readings were below the limit of detection and could not be reliably quantified at the recommended assay dilution. As a result, T4 levels could not be reported.

In the correlation analyses, when combining the control group with the DIO (Supplementary Table 1), T3 correlated positively with dopamine levels in the hypothalamus (rho = 0.673, *p* < 0.05).

### Other hormones

**Fig. 4:**
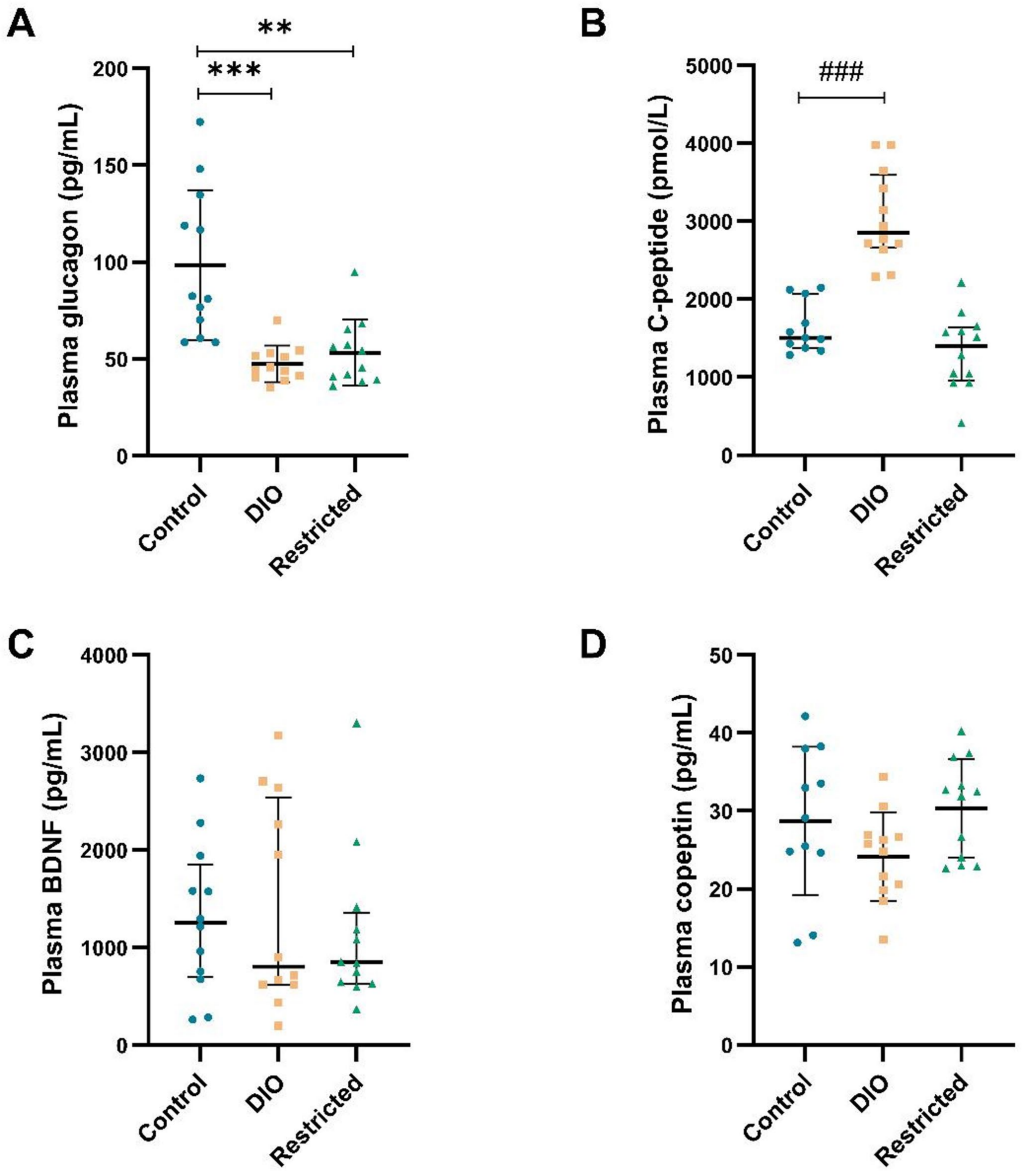
Plasma glucagon **A**, C-peptide **B**, BDNF **C**, and copeptin **D** levels in rats, after 12 weeks of diet for the control, diet-induced obesity (DIO), and restricted groups (*n* = 11–12/group). Data represent mean ± SD for **A** and **D**, and median with IQR for **B** and **C**. ** *p* < 0.01; *** *p* < 0.001 (t-test) and ### *p* < 0.001 (Mann-Whitney U test) compared to the control group

Glucagon levels were significantly lower in both the DIO and restricted group (*p* < 0.001; *p* < 0.01, respectively) compared to the control group (Fig. 4A). C-peptide levels were significantly higher for the DIO group (*p* < 0.001), but did not differ between the restricted group and the controls (Fig. 4B). No group differences in copeptin or BDNF levels were revealed compared to controls (Fig. 4C and D).

In the correlation analysis, when combining the control group with the restricted (Supplementary Table 2), copeptin inversely correlated with the dopamine levels in the nucleus accumbens core (rho = -0.699, *p* < 0.05) and with shelter-seeking behavior (rho = -0.668, *p* < 0.001).

### Multivariate data analysis

**Fig. 5:**
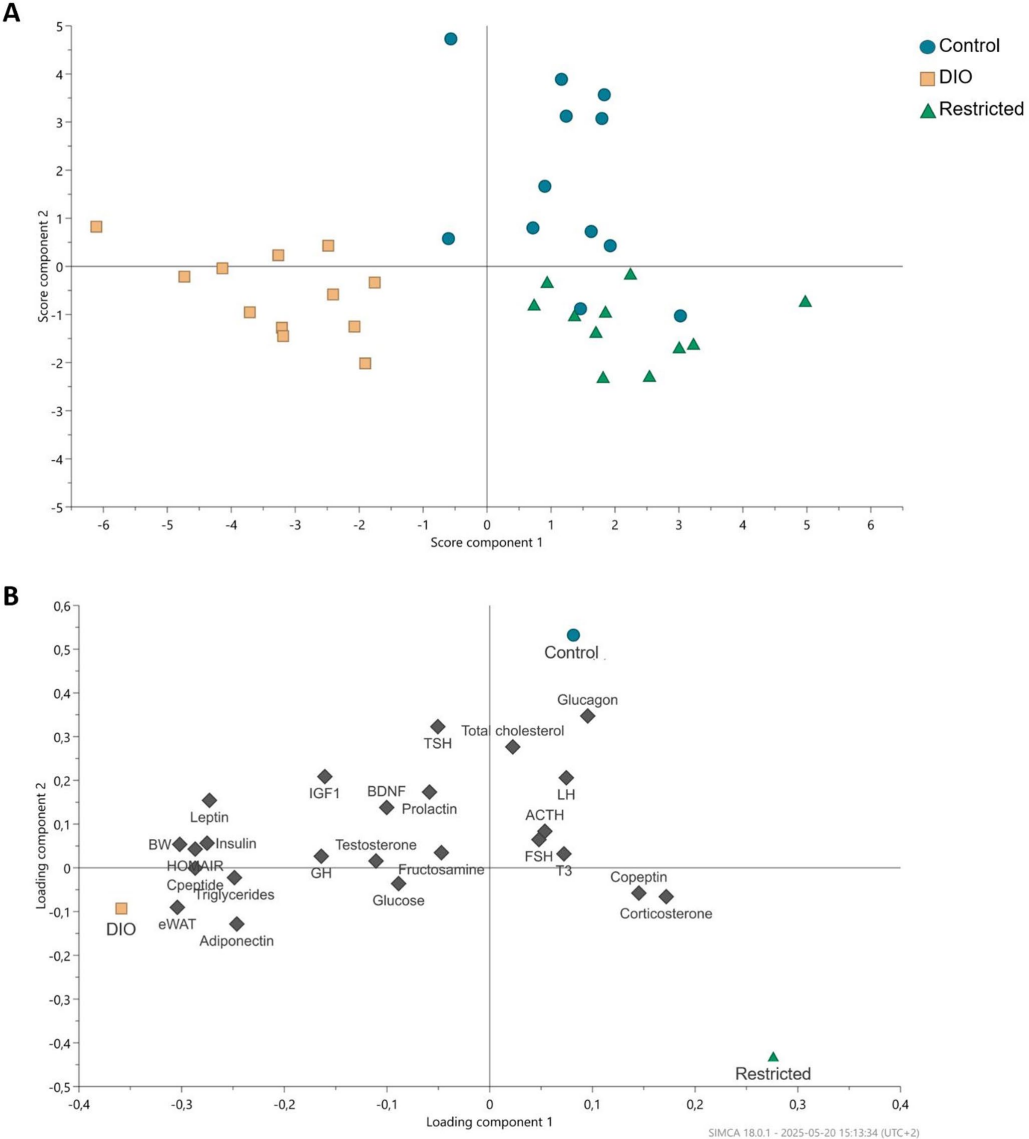
OPLS-DA score plot showing the individual rats **A** and the variable loading plot of the pituitary hormones, weight measurements and metabolic parameters **B** [21] after 12 weeks of diet for the control, diet-induced obesity (DIO) and restricted groups (*n* = 12/group; two significant components, R^2^X(cum) = 0.426, R^2^(cum) = 0.667, Q^2^(cum) = 0.555, R^2^Y(cum) = 1.000). Variables with high loadings were key contributors to the analysis, while variables close to the origin contributed less. Abbreviations: ACTH, adrenocorticotropic hormone; BDNF, brain-derived neurotrophic factor; BW, body weight; eWAT, epididymal white adipose tissue; FSH, follicle-stimulating hormone; GH, growth hormone; IGF-1, insulin-like growth factor 1; LH, luteinizing hormone; T3, triiodothyronine; TSH, thyroid-stimulating hormone.

OPLS-DA was performed to investigate group discrimination and identify the variables from the pituitary hormones and previously published metabolic parameters [21] that contributed most to the observed differences. The model resulted in two significant components with good explanatory and predictive ability, capturing 42.6% of the total variance in the predictor variable (R²X), 66.8% of the variance in the group classification (R²Y), and achieving a predictive ability of 55.5% (Q²). The OPLS-DA score plot (Fig. 5A) showed a clear separation between the experimental groups, particularly separating out the DIO group in comparison to the control and restricted groups. Two control rats showed a slight overlap with rats in the restricted group (Fig. 5A). The corresponding loading plot (Fig. 5B) revealed which variables contributed to the group separations. Body weight, epididymal white adipose tissue (eWAT) weight, metabolic markers (insulin, leptin, HOMA-IR, C-peptide, triglycerides, and adiponectin), and to a lower extent, IGF-1 and GH were of importance for the loading of the DIO group. In support of the conventional statistics, with lower glucagon levels in the DIO and restricted groups, glucagon loaded close to the control group. Finally, the restricted group loaded opposite to leptin, IGF-1, and TSH.

## Discussion

This study aimed primarily to investigate how pituitary hormonal axes and their corresponding peripheral hormones were affected after a 12-week cafeteria- or calorie-restricted diet in male rats. In addition, differences in the weight of endocrine glands were evaluated, and correlations of the hormones with behavior parameters and dopamine levels in specific brain regions were explored. Since many of these hormones are altered during obesity and metabolic dysregulations in humans [18], the hypothesis was that the same would be observed in the DIO group. However, significant changes were observed in absolute and relative tissue weights, while only minor alterations of the pituitary hormonal axes and their corresponding peripheral hormones were observed in the DIO rats. In contrast, more pronounced effects were revealed for the restricted group compared to controls, with differences both in hormone levels and tissue weights. These findings contribute to further understanding of diet-induced effects on neuroendocrine regulation of metabolism in rats, highlighting both similarities and differences between rats and humans.

The HPA axis is important for coordinating the body’s response to stress. Herein, no differences in either ACTH or corticosterone levels were observed between the DIO and control groups. These results align with the absence of stress-related behavioral differences between the groups, as assessed by the MCSF [21]. One potential reason is that the anxiolytic effect of the cafeteria diet in comparison to other diets used in rat obesity models [19, 31] could counteract the anxiogenic effects of obesity, a phenomenon that has also been observed in rats exposed to chronic stress [32]. Another possible explanation is the hormonal adaptation that might have occurred after a 12-week dieting period. This is supported by previous findings in rats, where higher levels of ACTH were observed after 1 and 9 weeks of high-fat diet, but not after 12 weeks [33]. Notably, and in conflict with the observation herein, that was not the case for corticosterone, which remained at higher levels during the whole period [33], probably due to the different diets. Our group has previously shown that ACTH levels were not altered in humans in a fasting state in obese individuals with or without diabetes in comparison to normoglycemic lean individuals [34, 35]. Moreover, cortisol levels (the human equivalent of corticosterone in rats) were lower in individuals with obesity in one study [34] and unchanged in another study of individuals with and without obesity, prediabetes, and diabetes [35]. However, under dynamic conditions (i.e., during hypo- or hyperglycemic clamps), Lundqvist et al. (2023 [35]) demonstrated significantly greater HPA axis responses in obese individuals, showing that the observed dysregulation can remain undetected under non-stimulated conditions. Furthermore, the calorie-restricted group showed significantly lower ACTH levels, even though the corticosterone levels were numerically somewhat higher. This is in agreement with a previous study [36] and is possibly explained by an enhanced sensitivity of the adrenal gland to ACTH stimulation.

Obesity negatively impacts fertility in both sexes. However, evidence regarding male infertility due to obesity is contradictory [37]. In general, an altered gonadal hormone profile is observed in men with obesity, with higher estrogen levels and lower testosterone, FSH, and LH levels [37–40]. Our group has previously reported a negative correlation between testosterone levels and adiposity markers in male individuals with obesity [41], which was not observed herein. The DIO group had lower levels of LH, which is consistent with findings in humans [37, 38]. In contrast, testosterone levels did not differ compared to controls, which is in agreement with a previous study using an obesogenic diet in rats, where no differences in testosterone levels were observed between obese and control groups [42]. However, this contrasts with human studies [37] and also with findings in a genetically obese Zucker rat model, where lower testosterone levels were reported in the obese compared to the lean group [43]. This shows that different rodent models of obesity can have divergent phenotypes, which should be considered when linking rodent obesity models to human obesity.

A U-shaped relationship has been seen between prolactin levels and metabolic health, with both very high and very low levels of prolactin being associated with higher risk for metabolic syndrome and T2D in humans [44, 45]. Since dopamine acts as an inhibitory modulator on prolactin secretion [45], the lower levels of prolactin could have been explained by higher dopamine levels in the median eminence secreted from the tuberoinfundibular neurons located in the arcuate nucleus of the hypothalamus. The total hypothalamic dopamine content did not differ between the groups [30], and herein, prolactin and dopamine levels in specific brain regions were not correlated. However, dopamine levels or turnover in specific nuclei of the hypothalamus, such as the arcuate nucleus, could differ and be linked to the changed prolactin levels herein. It has also been seen that stimulation of the arcuate nucleus dopamine neurons leads to increased food intake [46]. In that context, we found an inverse relationship between prolactin and exploratory activity in the MCSF when the DIO and control groups were combined.

In both human obesity and DIO rat models, the highly pulsatile GH secretion is generally decreased [47, 48], while the effects on IGF-1, the major mediator and marker of GH action over time, and peripheral tissues have been far from concordant [49, 50]. Herein, no differences in either GH or IGF-1 levels were observed after cafeteria diet, but there were significantly lower levels of IGF-1 after caloric restriction. It has been hypothesized that lower IGF-1 due to caloric restriction could be a protective mechanism against metabolic syndrome [24, 51], but again, there have been conflicting data showing that caloric restriction did not lower IGF-1 levels in obese rats [50].

The HPT axis has an important physiological role, affecting different functions such as thermogenesis, energy expenditure, and regulating the balance between lipogenesis and lipolysis [52]. In general, it is reported that TSH levels are slightly higher in human obesity [52, 53], but this does not necessarily mean a dysfunction in the thyroid gland. Herein, neither TSH nor T3 was changed by the cafeteria diet, indicating that the HPT axis was unaffected. In animal studies, both higher and lower levels of TSH have been seen after a high-fat diet or a cafeteria diet, with T3 levels remaining unchanged by either diet [54, 55]. Herein, TSH levels, but not T3 levels, were significantly lower after caloric restriction, possibly acting as a protective mechanism to conserve energy during periods of limited energy intake. Those results are partly supported by previous studies, which found decreased levels of both TSH and T3 after caloric restriction in rats [56, 57].

Lastly, a significantly lower weight of the pituitary gland in both the DIO and restricted groups was observed, mainly due to the change in the anterior lobe weight. However, when adjusted for body weight, this was true for the DIO rats only. This might be due to a negative trophic effect of DIO on pituitary growth and development, a finding that, to our knowledge, has not been previously reported. However, despite these changes, the pituitary-periphery hormonal axes seemed unaffected.

The multivariate data analysis included pituitary hormone measurements along with data on body weight, eWAT weight, and metabolic markers from the previous study [21]. The OPLS-DA revealed a clear separation between the groups, especially the DIO group separated from controls and the restricted group. The separation of the DIO group was driven mainly by body weight and eWAT weight measurements and metabolic markers, but with a contribution also from IGF-1 and GH levels. Even though changes may not reach statistical significance when hormones are assessed separately, differences can still contribute to the observed group separations. Moreover, the lower levels of IGF-1 and leptin [21] in the restricted group could contribute to the loading of the restricted group in the opposite quadrant. This suggests that while caloric restriction potentially leads to significant changes in a few specific hormonal axes, particularly those related to stress, growth, and thyroid function, the cafeteria diet may induce a more widespread shift in the energy homeostasis and glucose and lipid metabolism.

### Strengths and limitations

The assessment of the majority of the anterior pituitary hormones gives insights into how specific dietary interventions may lead to adaptations in neuroendocrine pathways, which in turn can contribute to the peripheral hormonal, metabolic, and other functional alterations. This might both directly and indirectly affect metabolism and some other key functions in the periphery. Furthermore, the use of both cafeteria diet, ad libitum fed controls, and caloric restriction allows for a comprehensive assessment of the brain’s adaptation during different dietary interventions, also given the fact that ad libitum fed controls have been questioned [58].

The non-standardized blood samplings [21], i.e., time of the day and fasting/fed, which differed between calorie-restricted and the two other groups, are a limitation of the study that needs to be taken into consideration. This can affect levels of some of the studied hormones and also metabolic measures, and the effects of calorie-restriction must be interpreted with caution. Moreover, the sampling time in the light/dark cycle is not identical between groups, and the assessment of the hormones are during a single time point and not a dynamic response, which limits the interpretation regarding responsiveness of pituitary axes. Finally, the use of only male animals is another limitation, as results are not generalizable to both sexes. Thus, future animal studies should be conducted in both sexes.

## Conclusion

We did not observe a clear dysregulation of neuroendocrine pathways following the high-caloric cafeteria diet, which was contrary to our hypothesis. In contrast, caloric restriction led to clear changes in several pituitary hormonal axes. Of note, the relative weights of several endocrine glands were decreased in DIO rats. Although this model has some similarities, e.g. regarding metabolic dysregulation, with early stages of human obesity and prediabetes, it primarily addresses the effects of overfeeding per se. Altogether, it yields a quite different phenotype than what is typical for human obesity, which is a multifactorial condition influenced by social, mental, behavioral, neurohormonal and other determinants.

## Supplementary Information

Below is the link to the electronic supplementary material.

Supplementary Material 1

## Data Availability

Some or all datasets generated and/or analyzed during the current study are not publicly available but are available from the corresponding author on reasonable request.
